# Bacteriophage-mediated lysis supports robust growth of amino acid auxotrophs

**DOI:** 10.1038/s41396-023-01452-7

**Published:** 2023-06-15

**Authors:** Gordon J. Pherribo, Michiko E. Taga

**Affiliations:** Department of Plant & Microbial Biology, University of California, Berkeley, Berkeley, CA 94720 USA

**Keywords:** Bacteria, Bacteriophages, Bacterial evolution, Bacterial secretion

## Abstract

Microbial communities host many auxotrophs—organisms unable to synthesize one or more metabolites required for their growth. Auxotrophy is thought to confer an evolutionary advantage, yet auxotrophs must rely on other organisms that produce the metabolites they require. The mechanisms of metabolite provisioning by “producers” remain unknown. In particular, it is unclear how metabolites such as amino acids and cofactors, which are found inside the cell, are released by producers to become available to auxotrophs. Here, we explore metabolite secretion and cell lysis as two distinct possible mechanisms that result in the release of intracellular metabolites from producer cells. We measured the extent to which secretion or lysis of *Escherichia coli* and *Bacteroides thetaiotaomicron* amino acid producers can support the growth of engineered *Escherichia coli* amino acid auxotrophs. We found that cell-free supernatants and mechanically lysed cells provide minimal levels of amino acids to auxotrophs. In contrast, bacteriophage lysates of the same producer bacteria can support as many as 47 auxotroph cells per lysed producer cell. Each phage lysate released distinct levels of different amino acids, suggesting that in a microbial community the collective lysis of many different hosts by multiple phages could contribute to the availability of an array of intracellular metabolites for use by auxotrophs. Based on these results, we speculate that viral lysis could be a dominant mechanism of provisioning of intracellular metabolites that shapes microbial community structure.

Microbial communities are ubiquitous, contributing to global nutrient cycling, human health and agricultural productivity. Nutritional interdependence is a universal feature of microbial communities [1]. One form of nutritional interaction occurs between auxotrophs—organisms unable to produce a nutrient required for growth—and prototrophs or “producers”—organisms capable of synthesizing a nutrient required by others. Auxotrophy contributes to microbial community stability [2, 3], and many bacteria are predicted to be auxotrophic for one or more amino acids or cofactors [4, 5], likely because auxotrophy often confers a growth advantage when the required nutrient is available [5]. The prevalence of auxotrophy suggests that a consistent source of nutrients must be available over evolutionary timescales, yet it is unknown how metabolites that function intracellularly, such as amino acids and cofactors, become accessible to auxotrophs. We previously proposed that nutrient release resulting from secondary effects of evolved biological processes, such as secretion of excess metabolites or cell lysis, are possible mechanisms of nutrient provisioning [6]. Here, we experimentally test both mechanisms using two phylogenetically distinct producer species and engineered *Escherichia coli* amino acid auxotrophs.

Amino acids and dipeptides have been found in exometabolomes of cultured microorganisms and soil biocrusts [7, 8]. Furthermore, certain pairs of amino acid auxotrophs can grow in coculture in the absence of amino acid supplementation, suggesting that bacteria may shed amino acids during growth [4, 9, 10]. To test whether amino acid secretion or leakage can be sufficient to support the growth of auxotrophs, we measured the extent to which cell-free supernatants from *E. coli* and *Bacteroides thetaiotaomicron*, both producers of all 20 proteogenic amino acids, could support the growth of up to 11 different *E. coli* amino acid auxotrophs in the absence of amino acid supplementation. Supernatants from both *E. coli* and *B. thetaiotaomicron* minimally supported most of the tested amino acid auxotrophs used in this study, with each producer cell supporting the growth of less than 0.2 auxotroph cells in all except the Trp auxotroph (Fig. 1A, B). Amino acid release was only slightly influenced by growth phase, as cell-free supernatants of producers harvested in stationary (Fig. 1) or exponential phase (Fig. S1) support only modest levels of auxotroph growth. Together, these results suggest that secretion or leakage may not be dominant forms of amino acid provisioning.

**Fig. 1:**
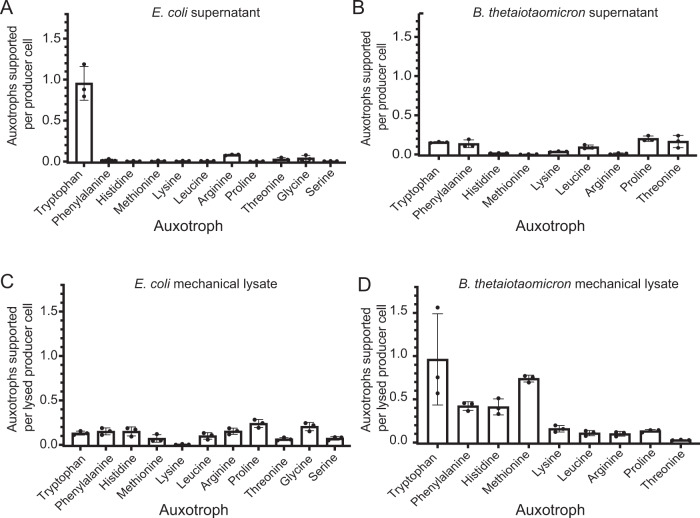
Cell-free supernatants and mechanical lysates minimally support growth of amino acid auxotrophs. The number of auxotroph cells supported per producer cell was calculated for auxotrophs grown in defined medium containing (**A**, **B**) cell-free supernatants and (**C**, **D**) French pressed lysates of (**A**, **C**) *E. coli* and (**B**, **D**) *B. thetaiotaomicron*. The auxotrophs are ordered from left to right on the x-axis based on the estimated biosynthetic cost of producing one molecule of the amino acid [4]. The genotype of each auxotroph is listed in Supplementary Table 1. In all except one case (Met), a single gene in the biosynthetic pathway is deleted [4]. Bars represent the mean of three replicates; error bars represent standard deviation.

We explored the possibility that cell lysis can release amino acids at sufficient levels to support auxotrophs. Lysis occurs naturally in bacteria following exposure to toxins or as part of developmental genetic programs such as sporulation in *Bacillus subtilis* and fruiting body formation in *Myxcococcus xanthus* [11–13]. A common trigger of microbial lysis is viral infection, as viruses are abundant and ubiquitous across many environments [14]. In marine environments, viruses lyse an estimated 20% of microbial biomass per day and are major drivers of microbial turnover and elemental cycling, a process known as the viral shunt [15, 16]. Further, in a laboratory coculture, addition of a lytic phage can lead to increased growth yield of a partner strain [17, 18].

To determine whether the levels of intracellular amino acids liberated via lysis of a producer could be sufficient to support the growth of amino acid auxotrophs, we tested the ability of the 11 amino acid auxotrophs to grow in media supplemented with mechanically lysed *E. coli* or *B. thetaiotaomicron* cells. Lysates of both producers contain a higher level of bioavailable amino acids than their respective supernatants, though less than one auxotroph cell was supported by each lysed producer cell (Fig. 1C, D). Comparison of lysates of *E. coli* and *B. thetaiotaomicron* suggests that these bacteria contain different levels of several amino acids, which likely reflects their distinct metabolic characteristics. The strains that were most supported by mechanical lysates of *B. thetaiotaomicron* were those auxotrophic for the more biosynthetically costly amino acids (Fig. 1D) (in all graphs, the auxotrophs are ordered based on their estimated biosynthetic cost [4]). Yet, with less than one auxotroph cell supported for each lysed producer cell, the ratio of producer to auxotroph cells in a community would need to be high.

During infection, viral energy demands are met by reprograming their host’s metabolism by overexpressing translational genes and elevating levels of intracellular metabolites [19, 20]. We therefore tested whether producers lysed by a phage could support auxotroph growth to a greater extent than mechanically lysed cells. Indeed, *E. coli* T4*rI* phage lysates, filtered to remove phage particles, supported the growth of all 11 auxotrophs at levels higher than supernatants and mechanically lysed *E. coli*, with nine of the 11 auxotrophs at a level exceeding one auxotroph supported by one producer (Fig. 2A). Five auxotrophs were supported at levels ranging from 11 to 47 auxotrophs per producer, suggesting that producers and auxotrophs can coexist at a low ratio (Fig. 2A). Similarly, *E. coli* λ*vir* lysates provided high levels of certain amino acids, supporting as many as 27 auxotrophs per producer cell (Fig. 2B). Upon observing that the two *E. coli* phage lysates showed differences in the levels of bioavailable amino acids, we compared these results with nine auxotrophs treated with lysates of *B. thetaiotaomicron* infected with the lytic phage ФSJC12 and found they supported auxotroph growth to a higher degree than *B. thetaiotaomicron* supernatants or mechanical lysates with, in three cases, over 30 auxotroph cells supported per producer cell (Fig. 2C). Together, these results demonstrate that phage lysis contributes a higher level of most amino acids compared to mechanical lysis or secretion; different phage infections and host cells vary in the levels of bioavailable amino acids released upon lysis; and in general, biosynthetically costly amino acids are released at higher levels than less costly amino acids. Though these results are limited to two producer bacteria and three phages in artificial conditions, they suggest that infection of diverse bacteria with different phages could support a variety of auxotrophs in microbial ecosystems. We postulate that phage lysis may be a dominant mechanism of intracellular nutrient release, potentially shaping the nutritional environment and contributing to the evolution of auxotrophy.

**Fig. 2:**
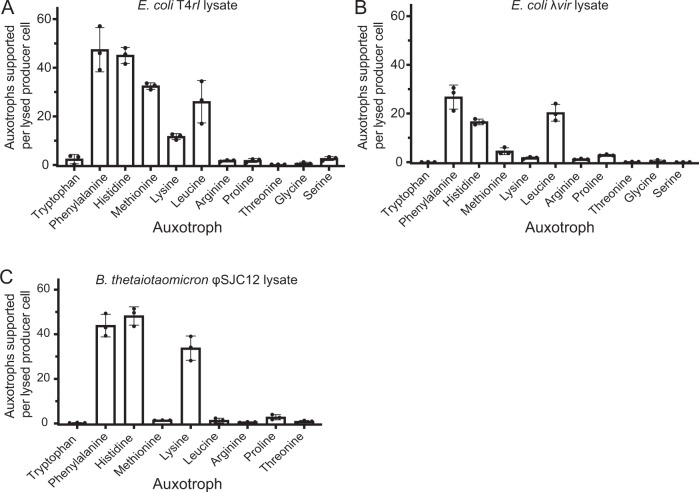
Phage lysates robustly support growth of certain amino acid auxotrophs. The number of auxotroph cells supported per producer cell was calculated for auxotrophs grown in defined medium containing (**A**) *E. coli* T4*rI* phage lysate, (**B**) *E. coli* λ*vir* lysate and (**C**) *B. thetaiotaomicron* ϕSJC12 lysate. The amino acids are ordered as described in Fig. 1. Bars represent the mean of three replicates; error bars represent standard deviation.

## Supplementary information

Supplemental Material

## Data Availability

The datasets generated and analyzed during the current study are available from the corresponding author on reasonable request.
